# Toward an Improved Understanding of Dyslexia: Reflections on a New Consensus Definition and Its Implications

**DOI:** 10.1002/dys.70022

**Published:** 2025-12-15

**Authors:** Kinga Morsanyi, Silvia Paracchini, Saloni Krishnan, Catherine Manning, Jennifer Milne, Jo Van Herwegen, Michelle Luciano

**Affiliations:** ^1^ Department of Mathematics Education Loughborough University UK; ^2^ School of Medicine University of St Andrews UK; ^3^ Psychology and Language Sciences University College London UK; ^4^ School of Psychology University of Birmingham UK; ^5^ School of Humanities, Social Sciences and Law University of Dundee UK; ^6^ Department of Psychology and Human Development, Institute of Education University College London UK; ^7^ Department of Psychology University of Edinburgh UK

**Keywords:** commentary, Delphi study, dyslexia definition, dyslexia diagnosis, intervention, multifactorial approach

## Abstract

Inconsistencies in the definition and diagnosis of dyslexia continue to impede research, assessment, and intervention. This paper, authored by members of the UK Specific Learning Difficulties Network, critically examines a recent effort to establish a consensus definition and guidance for assessment and intervention for dyslexia, which involved 58 experts from academia, practice and lived experience. The resulting framework offers a developmental, multifactorial and continuum‐based perspective. It expands on prevailing definitions by highlighting the influence of multiple genetic, environmental and cognitive factors, as well as developmental changes in dyslexia profiles. However, unresolved issues remain, including ambiguity around prevalence and the role of general cognitive functioning, and a lack of clear guidance for educators. The definition also lacks clarity on assessment and intervention, especially in global contexts where teaching practice may be highly variable. Co‐occurrence with language and mathematics difficulties is acknowledged but underexplored, limiting implications for practice. Whilst the Delphi method provides useful consensus, we also reflect on its limitations, including potential bias in the composition of the panel. Overall, the framework is a valuable step forward, but unresolved issues remain both from a research perspective and in terms of practical implementation.

## Practitioner Points

- We comment on recent attempts to establish consensus around the definition of dyslexia and how it should be assessed.
- The new definition highlights the role of multiple genetic, environmental, and cognitive factors in dyslexia and changes across development.
- Reading fluency is considered a core indicator of dyslexia across languages, but other cognitive factors are also highlighted.
- Processing difficulties are proposed to be linked to dyslexia, but it is not clear how practitioners should test these.
- Despite the merits of the new definition, it is not straightforward to translate it into guidance for practitioners.

The multiple definitions currently in use for dyslexia lead to inconsistency in assessment and identification (Sadusky et al. 2022) and hinder research into the condition, with different research groups studying slightly different populations. A study that has aimed to establish a consensus definition of dyslexia (Carroll et al. 2025) and how it should be assessed and identified (Holden et al. 2025) therefore represents a worthy goal, which may help us move beyond some current controversies and misinformation. In this paper, we (members of the UK Specific Learning Difficulties Network)^1^ offer an evaluation of the proposed definition of dyslexia and related guidance for assessment, as well as the methodology used to develop these, examining both their strengths and limitations. The paper seeks to highlight the key contributions of the new framework, particularly its adoption of a multifactorial and developmental perspective and its potential to unify practice across research, education, and clinical domains. However, we also comment on a number of unresolved issues, including ambiguities around the recommended diagnostic criteria and assessment methods, as well as the role of co‐occurring conditions in the diagnostic process and the planning of interventions. Drawing on perspectives from neurobiology, genetics, developmental psychology and educational practice, we aim to enrich the interpretation of the consensus framework and evaluate its relevance for diverse professional audiences.

## 1 The Delphi Procedure and Outcome

The definition and recommendations presented by Carroll et al. (2025), and Holden et al. (2025) are based on a Delphi study, which is a commonly used method to obtain input from a group of experts in a structured and iterative way (e.g., Hsu and Sandford 2007). In the Delphi process, after providing initial responses, participants may adjust their ratings based on feedback from the group in a number of subsequent iterations. Although typically, the aim is to elicit consensus on a topic, an alternative aim may be to obtain a broad range of opinions (cf., Diamond et al. 2014).

In the present case, the aim was to achieve consensus by conducting a two‐stage Delphi study with 58 dyslexia experts (57 in the second round), including academic researchers, educational psychologists, specialist teachers and assessors, representatives of dyslexia organisations, and individuals with lived experience of dyslexia. Panel members were presented with 55 statements relating to dyslexia, which they had to rate from “Strongly disagree” to “Strongly agree”, or “No opinion/Do not know”. A threshold for consensus was set at an overall agreement rate of 80%, which did not include the “No opinion/Do not know” responses. Overall, 42 statements achieved 80% consensus after the two survey rounds, and nine of these statements formed the basis of the proposed definition of dyslexia (Table 1). The statements were also used to develop updated guidance on assessment and intervention (see Carroll et al. 2025; Holden et al. 2025 for more details).

**TABLE 1:** Definitions of dyslexia by Rose (2009) and Carroll et al. (2025).

Definition of dyslexia by Rose (2009)	The statements included in the proposed definition of dyslexia by Carroll et al. (2025)
Dyslexia is a learning difficulty that primarily affects the skills involved in accurate and fluent word reading and spelling. Characteristic features of dyslexia are difficulties in phonological awareness, verbal memory and verbal processing speed. Dyslexia occurs across a range of intellectual abilities. It is best thought of as a continuum, not a distinct category, and there are no clear cut‐off points. Co‐occurring difficulties may be seen in aspects of language, motor coordination, mental calculation, concentration and personal organisation, but these are not, by themselves, markers of dyslexia.	Dyslexia is a set of processing difficulties that affect the acquisition of reading and spelling. In dyslexia, some or all aspects of literacy attainment are weak in relation to age, standard teaching and instruction, and level of other attainments. Across languages and age groups, difficulties in reading fluency and spelling are a key marker of dyslexia. Dyslexic difficulties exist on a continuum and can be experienced to various degrees of severity. The nature and developmental trajectory of dyslexia depend on multiple genetic and environmental influences. Dyslexia can affect the acquisition of other skills, such as mathematics, reading comprehension or learning another language. The most commonly observed cognitive impairment in dyslexia is a difficulty in phonological processing (i.e., in phonological awareness, phonological processing speed or phonological memory). However, phonological difficulties do not fully explain the variability that is observed. Working memory, processing speed and orthographic skills can contribute to the impact of dyslexia. Dyslexia frequently co‐occurs with one or more other developmental difficulties, including developmental language disorder, dyscalculia, ADHD and developmental coordination disorder.

## 2 Comments on the Definition of Dyslexia

The current prevailing definition of dyslexia, used by many UK‐based dyslexia assessors, is the definition offered by Rose (2009) (Table 1).

Although there are no points on which Carroll et al. (2025) would clearly conflict with Rose (2009), the new definition and guidance go beyond this earlier conceptualisation in multiple ways. One of these is that it takes a developmental perspective by proposing that the manifestations of dyslexia change over time, for example, as a result of instruction, intervention, and support. Carroll et al. (2025) also use this as an argument against using predetermined cut‐off points in the diagnostic process. Whilst persistence of difficulties is emphasised, they recommend that this should be considered from a developmental perspective.

The proposed approach also goes beyond Rose by advocating for a multifactorial view of dyslexia, making references to multiple genetic, environmental, and cognitive factors that can impact developmental trajectories in reading and spelling skills. Even though phonological skills are emphasised, Carroll et al. (2025) reject the view that a single cause (such as weak phonology or problems with working memory) may underlie dyslexia. On this point, they concur with Rose, who also advocated for multiple cognitive factors to be considered. Whilst Carroll et al. (2025) retain the idea of unexpectedness of difficulties, the proposed approach offers more guidance on how this should be interpreted. For example, if a person has an intellectual disability, reading difficulties are no longer unexpected. Conversely, resistance to intervention or slower school progress in reading and spelling than in other areas of learning can offer evidence of unexpectedness. The new approach also highlights the importance of reading fluency (which is defined as accurate and automatic reading). Reduced fluency may be a very useful indicator of dyslexia, especially in the case of adults, for whom some aspects of reading attainment may be in the typical range.

An additional contribution of Carroll et al. (2025) is that they address some common assumptions and misconceptions relating to dyslexia. Specifically, they distinguish between developmental and acquired dyslexia, as well as dyslexia and visual stress. They also state that left‐ or mixed‐handedness is not useful in identifying dyslexia, and they also do not support the claim that dyslexia confers advantages in creative or visual–spatial skills.

Whereas the new definition offers useful extensions and clarifications, it leaves several issues unresolved. For example, by advocating a continuum view, it does not give an indication of prevalence, which would be useful in supporting identification, as well as in allocating resources for assessment, support, and intervention. Whilst not using specific cut‐offs may make sense in a clinical setting, in a research context, this can be problematic, as it leads to variations in dyslexic samples across studies (Sadusky et al. 2022). One approach to resolve this issue may be to rely on samples with an existing dyslexia diagnosis, although this method has its disadvantages given the underdiagnosis of dyslexia (e.g., Barbiero et al. 2019), and under‐representation of parts of the population in dyslexia diagnosis (Knight and Crick 2021; Strand and Lindorff 2021). Another option (which is very often used in research) is to rely on reading assessments with specific cut‐offs, although this goes against the recommendations of Carroll et al. (2025), and the continuum view. A potential solution may be combining existing diagnosis or self‐identification with standardised assessments (e.g., Nalavany et al. 2018), which aligns better with the recommendations of Holden et al. (2025); see below.

The new definition also does not resolve issues around using a discrepancy criterion between reading and general cognitive functioning. It is notable that the ICD‐11 (WHO 2022) diagnostic guidance uses a discrepancy criterion relative to age and IQ, whereas the DSM‐5‐TR (American Psychiatric Association 2022) definition focuses on unexpected levels of attainment relative to age and educational opportunities. Carroll et al. (2025) offer somewhat confusing guidance relating to this by stating that although a discrepancy between intellectual ability and literacy could be a useful indicator of dyslexia, in itself, it is not sufficient for a dyslexia diagnosis. Nevertheless, they do not comment on whether it is a *necessary* condition. They also do not refer to the magnitude of discrepancy that could be relevant, in line with their approach to avoid recommending specific cut‐off points. There is further reference to levels of general cognitive functioning in that they propose that when an individual has an intellectual disability, a dyslexia label may result in a too narrow approach to intervention. Nevertheless, they maintain that all individuals with literacy difficulties should be offered targeted intervention, monitoring, and resources. Despite these references to general cognitive functioning, there is no clear guidance on whether this should be considered as part of a diagnostic assessment.

Carroll et al. (2025) also make no specific reference to stealth dyslexia, a term used to describe individuals who have high cognitive abilities and compensate for their dyslexia to varying degrees, so that they may not meet the criteria for assessment and diagnosis (Kranz et al. 2024). These individuals may be gifted or high‐achieving, so their underlying phonological difficulties are masked, and, outwardly, their reading abilities are often on par with their peers. Nevertheless, they usually have other predisposing factors, such as a family history of dyslexia. Spelling tends to be a main marker, together with word‐level reading and reading fluency, and can be significantly lower than other aspects of attainment as they progress through school. These learners, although not considered in the proposed framework, would benefit from appropriate intervention and support to prevent academic disengagement and underperformance, as well as potential mental health issues.

## 3 Comments Relating to Assessment

One of the aims of a consensus definition of dyslexia is to improve the process of identification and, ultimately, support. The revised definition offers several advantages in this respect as compared to the Rose (2009) criteria. First, it offers firmer direction in how dyslexia indicators should be viewed across languages and across age, highlighting difficulties in reading fluency and spelling as key markers. Second, bringing into the definition the knowledge that both genetic and environmental influences on dyslexia are important should direct assessors to collect relevant information in the early stages of assessment, with family history even serving as a key indicator in the information‐gathering stage.

Third, the revised definition now allows for some, but not necessarily all, aspects of literacy attainment to be weak in relation to expectations. This represents a more inclusive framework, which enables identification of dyslexia across a range of cognitive profiles and age groups and, importantly, might result in more targeted intervention and support, depending on a person's specific profile. This approach resolves some issues inherent in using cut‐offs in the diagnostic process, as recommended by the ICD‐11 (WHO 2022) and DSM‐5‐TR (American Psychiatric Association 2022), and aligns better with the possibility of changes in reading attainment with age and as a result of instruction or intervention. This ensures that the diagnostic criteria may still be met, even when a person shows improvement in some aspects of their reading. This is important, as without support, these individuals may fall behind their peers again (Thompson 2021). Consistent with the above, Holden et al. (2025) also recommend that, in the case of adults, it is important to establish evidence for early and persisting difficulties, even if these difficulties have been compensated for or masked. Nevertheless, they also allow for age‐related change in the opposite direction, where difficulties may surface when compensatory strategies no longer work. Thus, by emphasising the role of skilled professional judgement, which integrates background information, standardised assessments, and qualitative observations, the proposed approach could offer a chance to better establish any need for support and the specific level of support needed.

Fourth, more is said in this definition about the processing difficulties that are typically observed in dyslexia. Phonological processing (awareness, memory and speed), as in past definitions, remains key, but now working memory, processing speed and orthographic skills are highlighted as contributors to the impact of dyslexia. Nevertheless, apart from phonological processing, these are not considered causes but may influence severity, so, with regard to assessment, they would be useful adjuncts to a standardised battery of literacy‐related tests, even though, of themselves, they are not markers of dyslexia. However, the non‐specificity of the constructs gives assessors no clear guidance around the tests to include.^2^ For instance, is verbal rather than visuospatial working memory likely to be more informative for assessment? Similarly, which tests of orthographic skills will be free from phonological processing influence?

Related to the lack of a clear steer on the range of cognitive tests that should be used to assess dyslexia, as discussed above, the revised definition does not refer to general cognitive functioning. This means that it will again fall on professional judgement whether weak literacy skills should be supported in the context of a specific learning difficulty or a more general learning difficulty. The final aspect of the definition is around co‐occurring conditions, which in the Rose (2009) definition were highlighted as not being markers of dyslexia. The revised definition is agnostic in that it simply states the frequent co‐occurrence of dyslexia with one or more other difficulties. This cautious approach is justified given that empirical research has not yet established the causes of this increased co‐occurrence. Nevertheless, it serves as an important reminder to assessors that a dyslexia assessment needs to consider difficulties beyond those in reading, spelling and writing that may require additional specialist assessment and support.

Whereas some details are missing from the definition and guidance that could be crucial in an assessment context, a strength of Holden et al.'s (2025) work is that they make some high‐level recommendations for best practice relating to assessment and intervention. For example, they recommend that all individuals with literacy difficulties should receive targeted intervention, monitoring and resources, regardless of their general intellectual functioning, and they also advocate for early and sustained intervention. In line with the multifactorial view of dyslexia and a hypothesis‐testing approach, they also argue for a comprehensive assessment, taking into account family history and response to intervention before diagnosis, instead of mechanically applying scores and cut‐offs. They also advocate for using qualitative observations together with standardised test results, whilst also considering measurement error and expectancy biases, and emphasise that the content of assessment should be aligned with its purpose.

Holden et al. (2025) also propose that diagnostic assessment should not be a precondition of putting intervention in place. In fact, if relevant information is available, response to intervention (particularly, lack of progress) should be considered as part of the diagnostic process. Nevertheless, Holden et al. (2025) note that a positive response to intervention should not exclude the possibility of a dyslexia diagnosis. Conversely, no response to intervention is not necessarily a sign of dyslexia, as interventions may not always be appropriate. Indeed, teaching approaches used at school may also contribute to difficulties in children. The authors also note that if a child has not received intervention, this should not exclude the possibility of a dyslexia assessment (so, intervention should not be a prerequisite). Holden et al. (2025) also make policy recommendations, including universal screening, progress monitoring, and a further assessment if response to intervention is poor.

Regarding the above points relating to progress within the classroom context and response to intervention, it is important to highlight that many children do not receive the dose, intensity and duration (see, e.g., Conn and Chan 2016) of structured literacy instruction in the classroom or in focused intervention to achieve the potential response and progress, which impacts most dyslexic and other at‐risk readers (Moats 1999). Teachers worldwide are lacking the complex pedagogical and content knowledge and skills, along with significant other barriers, to provide effective initial instruction and intervention (Kehoe and McGinty 2024; Milne and Topping 2025). Teaching practitioners also hold misconceptions about effective reading instruction and dyslexia, which are exacerbated by multiple definitions, causing a lack of focus on the necessary literacy provision that would improve reading outcomes for dyslexic learners (e.g., Seidenberg et al. 2020). Indeed, Van Herwegen et al. (2024) highlighted that whilst educators might be more familiar with dyslexia than with other conditions (e.g., dyscalculia), they endorsed more neuromyths related to dyslexia. Better teacher training for dyslexia has also been highlighted as a key priority for the dyslexia community (Manning et al. 2025).

Although Carroll et al. (2025, 1065) set out to provide “clear and workable guidelines for assessment, identification, and education policy”, similar to a lack of detail in terms of appropriate assessment materials, there is no clear message for policy‐makers on the structured literacy instruction and intervention needed in classrooms, so that classroom practitioners can understand how their instruction in the various components of reading can positively impact the underlying phonological and related difficulties and improve reading outcomes for dyslexic learners. Referring to “standard teaching” in the definition is problematic, due to the aforementioned lack of evidence base and the dramatic variability—within schools, regions, nations and worldwide—in standard instruction, assessment and intervention. Research unequivocally indicates that reading instruction worldwide is not of the quality that would address the needs of all learners and particularly dyslexic learners (e.g., Crawford et al. 2025). Thus, instead of assuming the provision of effective classroom instruction or intervention, it should be underlined that improvements in outcomes for dyslexic learners would come from an increase in teacher knowledge and skill and provision of high‐quality literacy instruction and intervention (e.g., Binks‐Cantrell et al. 2022).

## 4 Comments From a Neurobiology and Genetics Perspective

The revised definition of dyslexia largely omits discussion of its neurological basis. This is reasonable given that current neuroimaging tools are not suited for diagnosis. Nevertheless, dyslexia is fundamentally a neurobiological condition. Over the last two decades, neurobiological research has helped us understand how development, reading experience and language/writing systems shape the brain (Romeo 2023). Neural correlates of dyslexia are strikingly consistent across languages, even when behavioural profiles differ (Paulesu et al. 2001; Yan et al. 2021), and structural brain differences are detectable in children with a familial risk for dyslexia *prior* to the beginning of reading instruction (Turesky et al. 2025). More recently, large‐scale neuroimaging datasets linked polygenic risk scores to variability in language‐relevant brain regions, offering a mechanistic bridge between genetic risk and behavioural outcomes (Carrión‐Castillo et al. 2023; Krishnan and Singh 2023). Intervention studies further reveal the malleability of key hubs in the brain's reading network, demonstrating that intensive instruction can shape neural pathways (Mitchell et al. 2025).

Despite these promising developments, one challenge is that whilst neural differences appear robust at the group level, these differences do not characterise every individual with reading difficulties. When studying a cohort based on needs but not diagnosis, there was no simple one‐to‐one mapping between brain structures and diagnostic labels. Instead, distributed and overlapping neural patterns were related to cognitive profiles (Astle et al. 2022). This indicates that, although group‐level consistency informs our understanding of the neurobiology of dyslexia, diagnosis and intervention design cannot rely on a single “brain signature”. Instead, recognising multiple potential neural pathways reinforces the value of profiling cognitive strengths and weaknesses to guide targeted intervention.

A practical consequence of adopting a broader approach to defining dyslexia is that it facilitates recruitment of larger, more representative samples, which, in turn, makes it possible to capture and analyse the full range of neurobiological and cognitive heterogeneity in dyslexia, rather than focusing on “pure” cases only. The new consensus definition therefore offers valuable opportunities to explore neurobiological distinctions between subgroups—for example, between dyslexic children with and without co‐occurring language difficulties or mathematics problems. With sufficiently large and diverse samples, imaging studies can also test mechanistic theories—for example, whether reading difficulties stem from impoverished phonological representations or from reduced access to these representations (Boets et al. 2013). This could advance our understanding of individual differences within the dyslexic population.

By recognising the co‐occurrence of dyslexia with other neurodevelopmental conditions and the variability in its manifestations, the definition also aligns with recent advances in genetic research. Historically, genetic studies of dyslexia often relied on strict inclusion and exclusion criteria. Participants were typically included based on single‐word reading measures (Gialluisi et al. 2021), whilst those with co‐occurring conditions such as ADHD or language impairments were excluded (e.g., Scerri et al. 2010). These practices were rooted in the assumption that isolating “pure” cases of dyslexia would make it easier to identify specific predisposing genetic factors. However, this approach, which required detailed assessment, made it difficult to recruit the large sample sizes needed for genome‐wide association studies (GWAS). The largest GWAS reported for dyslexia so far (*N* of about 50,000 cases and more than one million controls) has demonstrated a highly polygenic nature for dyslexia (Doust et al. 2022). This study represents a real breakthrough in the field and was made possible by the availability of a very large sample of dyslexia cases identified through self‐reported diagnosis and no strict inclusion/exclusion criteria. Furthermore, follow‐up studies have shown dyslexia to genetically correlate with other neurodevelopmental conditions like ADHD (Ciulkinyte et al. 2025), although this may be driven by cases in the GWAS samples with co‐occurring dyslexia and ADHD, which necessitates further research that compares single versus co‐occurring neurodivergence. By moving away from narrow diagnostic criteria, the new definition aligns with the picture of a highly complex and polygenic trait that is emerging from genetic studies. The question remains whether a difficulty restricted to the reading domain can be pinpointed to specific neurobiological factors. Therefore, whilst a neurobiological component was not directly embedded in the new definition, a broader approach has the potential to contribute to the study designs that could lead to a deeper understanding of dyslexia and its relationship within the broader spectrum of neurodevelopmental conditions.

## 5 Comments From the Perspective of Other Conditions

As described above, the new definition acknowledges that dyslexia frequently co‐occurs with one or more other neurodevelopmental conditions. This is in line with the genetic‐ and neuroscience evidence reviewed above, and broader findings that the co‐occurrence of neurodivergent conditions is more common than can be expected on the basis of chance alone, and it can be considered the rule rather than the exception (e.g., Dewey 2018). Nevertheless, the implications of these co‐occurrences for dyslexia diagnosis and intervention are not discussed by Carroll et al. (2025) or Holden et al. (2025). In this section, we reflect on this issue from the perspective of language‐ and mathematical difficulties in dyslexia.

The consensus statement noted that oral language skills and reading ability are closely intertwined, with (1) recognition that Developmental Language Disorder (DLD) frequently co‐occurs with dyslexia; (2) poor language skills confer additional risk for dyslexia, and, conversely, better language may be protective; and (3) dyslexia might limit opportunities for further growth in language. This recognition is laudable, and opens up several important directions for practice and research. It is clear that language and reading abilities substantially overlap—about half of the children diagnosed as dyslexic have oral language difficulties (McArthur et al. 2000). Yet, these children frequently receive limited to no support for their language, indicating that raising awareness of, and assessing for language challenges is paramount. This recommendation should be highlighted as being of immense importance to practitioners.

Second, the idea that good language skills may be protective indicates that a focus on language skills as well as reading during intervention could be beneficial, even if the child does not qualify for a diagnosis of DLD. Indeed, this focus on language skills could begin early, even prior to the onset of reading instruction, as a measure to reduce the risk of reading difficulties in the case of children with a family history of dyslexia. Finally, the recognition that dyslexia can affect the acquisition of later language skills is critical. After children start “reading to learn”, they primarily build their comprehension skills and vocabulary through reading, and by encountering more complex “book” language (e.g., Nation 2017). Yet, understandably, individuals with dyslexia enjoy reading less, which can establish a vicious cycle (Jones et al. 2025). Understanding how to break this cycle is an important goal for future research.

Carroll et al.'s (2025) definition of dyslexia also makes references to dyscalculia and mathematics learning difficulties. Beyond stating that dyslexia and dyscalculia often co‐occur, they also make a strong causal statement regarding the relations between dyslexia and mathematics difficulties, proposing that reading difficulties may impact on the acquisition of mathematics skills. It is important to reflect on these points, as no further commentary is offered by Carroll et al. (2025).

Whereas the relatively high co‐occurrence of dyslexia and dyscalculia is well‐documented (e.g., Haberstroh and Schulte‐Körne 2019), most cases of dyscalculia exist in isolation of reading difficulties (e.g., Morsanyi et al. 2018), and vice versa. Furthermore, individuals with dyscalculia or mathematics learning difficulties and dyslexic people with co‐occurring mathematics difficulties have distinct cognitive profiles. For example, a meta‐analysis (Szűcs 2016) showed that pupils with mathematics difficulties could be divided into two subtypes: those with reading problems and weak verbal working memory, and those without reading problems, but with weak visuo‐spatial working memory. Another study (Peters et al. 2020) found distinct cognitive profiles in dyslexia and dyscalculia, as well as an additive effect for co‐occurring dyslexia and dyscalculia, where the cognitive profile of children was similar to the sum of the impairments in the isolated dyslexia and dyscalculia groups. These findings make it clear that when mathematics difficulties are present in dyslexia, it is necessary to assess the possibility of dyscalculia as well, instead of assuming that mathematics difficulties are “simply” the result of reading problems. Moreover, it is important to provide interventions for mathematics difficulties independent of reading interventions, as it is very unlikely that reading interventions alone would resolve mathematics difficulties.

Even though we do not reject completely the possibility of causal relations between reading and mathematics skills, this may be specific to certain types of mathematics tasks, such as mathematical word problems (e.g., Vilenius‐Tuohimaa et al. 2008). In other cases, such as when problems with mental arithmetic are present in dyslexia, this may be better explained by specific problems with some of the underlying cognitive processes, such as phonological awareness (e.g., Yang et al. 2022) or verbal working memory (den Friso‐Van Bos et al. 2013), rather than by reading impairments, per se. Thus, to be able to provide appropriate intervention for mathematics difficulties, it is necessary to consider the broader cognitive profile of dyslexic learners, including the consideration of whether a diagnosis of dyscalculia would be appropriate. In turn, even when a general problem across various areas of mathematics is not present, which could be expected in the case of dyscalculia (Roulstone et al. 2024), specific interventions may still be recommended to avoid the negative consequences of low numeracy (e.g., Hudson et al. 2009).

## 6 Comments on the Delphi Methodology

The Delphi method can drive a research area forward by establishing whether opinions have moved on certain topics. Although the new definition does not offer radical changes to the Rose (2009) definition, it extends it in multiple ways and brings it in line with current theorising, which is an important contribution. An additional strength of the Delphi method is that it is iterative, which means that there are opportunities for refinement and clarifications; however, in the present case, only two iterations were used. It is also important that a predetermined criterion (i.e., achieving 80% agreement between panellists) was set before starting the study. High agreement can be useful in arguing for a change in different contexts, including education policy.

However, the method used also has its limitations. One of these is that, by focusing on agreement, there is a lack of attention to controversial issues, although Holden et al. (2025), briefly refer to a few areas of remaining controversy (e.g., the role of general cognitive functioning, whether cut‐off criteria should be used in assessment, to what extent dyslexia can be separated from reading problems in general, how to assess dyslexia in the case of additional language learners, and what causal factors and secondary factors are impacting prognosis). Moreover, by asking panellists to evaluate individual statements, there was little opportunity to integrate information across statements or to clarify seemingly self‐contradictory ideas. As a result, there are several examples of potentially self‐contradictory ideas in the definition: (1) assuming both persistence and developmental change; (2) indicating that interventions can be a protective factor, but claiming that the lack of progress despite intervention could be used as evidence for dyslexia; (3) not promoting a discrepancy criterion, but claiming that it could be useful; (4) recommending standardised testing without giving indications of how scores should be interpreted. Thus, despite the additional comments and interpretation, the message remains somewhat fragmented and unclear in some instances.

The intention to diversify the panel (i.e., in terms of languages used, geographic locations, gender, and background), and to move away from Anglocentric approaches, is laudable. Nevertheless, a question that arises is the extent to which the consensus definition reflects the views of the authors and their professional networks, as opposed to representing the full diversity of views across practitioners, academics and people with lived experience of dyslexia. Indeed, most panel members (73%) were from England and were females (71%), and a large proportion of them were academics (44%). Ensuring that the broadest perspectives possible are included is critical to ensure that the new definition really does represent a consensus that serves the whole dyslexia community. In this regard, whether consensus levels differed between stakeholder groups would have been informative, as well as presenting the results relating to selecting the “No opinion/Do not know” option together with levels of agreement. Indeed, given the small proportion (20%) of “other” stakeholders (i.e., beyond academics, educational psychologists and specialist teachers and assessors), their agreement was not necessary for reaching a consensus. Furthermore, when consensus was reached on a statement, it was not reported what proportion of panellists had contributed their ratings. Such data would be important in identifying knowledge gaps in the dyslexia community, and could be useful in identifying areas for future research, or for designing training and professional development materials. Eleven out of the 55 original statements were removed (see Holden et al. 2025 for a list). These could also be further considered in terms of future research questions and theorising.

## 7 Concluding Comments

The consensus definition of dyslexia by Carroll et al. (2025), alongside Holden et al.'s (2025) assessment recommendations, represents meaningful progress in reconciling theoretical, clinical, and educational perspectives on dyslexia. We particularly welcome the acknowledgement of changes over development in the manifestation of dyslexia and the multifactorial approach, both in terms of the origins of dyslexia and the associated cognitive profile. By adopting this framework, the proposed definition offers a more inclusive approach, which can support differentiated, needs‐based assessments and interventions whilst also opening up new avenues for research (see e.g., Astle and Fletcher‐Watson 2020). There is also a very clear message in terms of the importance of offering targeted reading intervention to struggling learners, preferably from a very early age, regardless of diagnostic status and level of general cognitive functioning.

Nevertheless, by broadening the definition there are further challenges to be addressed. The definition and recommendations seem to assume an ideal world, where all teachers are highly trained in reading instruction and interventions, and have good awareness of dyslexia and the underlying cognitive processes. Given that this is not the case, there is more information required on how the new definition can be used in practice and what assessments should be included to confirm a diagnosis of dyslexia (in different age groups), as well as recommendations for intervention approaches. These are all key elements of bridging the gap between theory and practice, and, ultimately, improving reading outcomes. Evidence reviews, such as the MetaSENse database^3^ may help with such efforts.

The absence of clear recommendations on co‐occurring language and mathematics difficulties also limits the practical utility of the proposed framework. Relating to this point, we stress the importance of, when relevant, offering separate assessment and targeted intervention for language and mathematics difficulties (as well as other co‐occurring conditions) for dyslexic learners. The lack of attention to neurobiological aspects of dyslexia also brings challenges around the specificity of dyslexia and how dyslexia differs from other reading difficulties, if indeed it does. A better understanding of overlaps and specificity of cognitive profiles across various neurodevelopmental conditions (Astle et al. 2022) has highlighted the overlap of domain‐general difficulties across various neurodivergences, which suggests that an approach focused on continuous dimensions of variation rather than distinct categories may be more fruitful in the future.

Whilst the Delphi method achieved high consensus across a broad range of issues, it did so with a panel heavily weighted toward academic and UK‐based professionals, raising concerns about the representativeness of the consensus. Casting a wide net and including a very large number of statements, as well as focusing on a consensus, also prevented the panel from engaging more deeply with some controversial issues. Whereas further insights could be gained from analysing the rejected statements and the qualitative comments of panel members, future Delphi studies could focus on some controversial or under‐explored issues (maybe with panel compositions matched to the specific topics), such as the role of general cognitive functioning in the diagnostic process and interventions, the importance of co‐occurring conditions, and dyslexia in additional language learners.

For this definition to achieve its full potential, future efforts must also bridge the gap between theory and practice. This includes implementing policy recommendations, enhancing educator guidance and support systems, and addressing inconsistencies in classroom instruction. Without such steps, the risk remains that even a scientifically sound definition will fail to transform educational experiences in dyslexia.

## Funding

This work was supported by the Medical Research Council (MR/X502716/1); Economic and Social Research Council (ES/W002914/1).

## Conflicts of Interest

The authors declare no conflicts of interest.

## Data Availability

Data sharing not applicable to this article as no datasets were generated or analysed during the current study.

## Endnotes

^1^
The Specific Learning Difficulties Network (SLDN; About Us | Learning Difficulties Network) brings together researchers from across the UK to coordinate the research agenda better and raise awareness of specific learning difficulties. By working with families, teachers, charities, and policymakers, the SLDN aims to make research useful to those with specific learning differences.

^2^
More detailed guidance for UK‐based assessors can be found on the SpLD Assessment Standards Committee website (Assessment Guidance).

^3^
MetaSENse | Centre for Educational Neuroscience.
